# Stereotactic Radiotherapy after Radical Prostatectomy in Patients with Prostate Cancer in the Adjuvant or Salvage Setting: A Systematic Review

**DOI:** 10.3390/cancers14030696

**Published:** 2022-01-29

**Authors:** Christina Schröder, Hongjian Tang, Paul Windisch, Daniel Rudolf Zwahlen, André Buchali, Erwin Vu, Tilman Bostel, Tanja Sprave, Thomas Zilli, Vedang Murthy, Robert Förster

**Affiliations:** 1 Institute for Radiation Oncology, Cantonal Hospital Winterthur (KSW), 8400 Winterthur, Switzerland; christina.schroeder@ksw.ch (C.S.); hongjian.tang@ksw.ch (H.T.); paul.windisch@ksw.ch (P.W.); daniel.zwahlen@ksw.ch (D.R.Z.); 2 Department of Radiation Oncology, Ruppiner Kliniken GmbH, Brandenburg Medical School (MHB), 16816 Neuruppin, Germany; a.buchali@ruppiner-kliniken.de; 3 Department of Radiation Oncology, Cantonal Hospital St. Gallen (KSSG), 9000 St. Gallen, Switzerland; erwin.vu@kssg.ch; 4 Department of Radiation Oncology, University Hospital Mainz, 55131 Mainz, Germany; tilman.bostel@unimedizin-mainz.de; 5 Department of Radiation Oncology, University Hospital Freiburg, 79106 Freiburg im Breisgau, Germany; tanja.sprave@uniklinik-freiburg.de; 6 Department of Radiation Oncology, University Hospital Geneva (HUG), 1205 Geneva, Switzerland; thomas.zilli@hcuge.ch; 7 Department of Radiation Oncology, Tata Memorial Hospital and Advanced Centre for Treatment Research and Education in Cancer (ACTREC), Homi Bhabha National Institute (HBNI), Mumbai 400012, India; vmurthy@actrec.gov.in

**Keywords:** prostate cancer, salvage, radiotherapy, toxicity, SBRT

## Abstract

**Simple Summary:**

Stereotactic body radiotherapy, a type of high-precision radiotherapy delivering high doses within few treatment sessions has proven to be effective and well tolerated in prostate cancer patients treated with definite radiotherapy. This systematic review summarizes the available data and analyzes whether this modern treatment may routinely be offered to prostate cancer patients after radical prostatectomy.

**Abstract:**

(1) Background: Prostate cancer is the most common cancer in men and can be treated with radical prostatectomy (RPE) or radiotherapy in the primary setting. Stereotactic radiotherapy (SBRT) has proven to be effective and well tolerated in this setting. However, if SBRT is an equally promising treatment option if applied in the adjuvant or salvage setting after RPE remains unknown. (2) Methods: We searched the PubMed and Embase databases with the following full-text queries in August 2021 for any combination of the terms “SBRT”, “prostate”, “adjuvant”, “postoperative”, “salvage”, “stereotactic radiotherapy”, “prostate bed”. There were no limitations regarding publication date or language. We adhered to the Preferred Reporting Items for Systematic Reviews and Meta-Analyses (PRISMA) recommendations. (3) Results: We identified 11 individual studies that were included in this systematic review. Three publications included patients without prior radiotherapy and the remaining eight patients with prior radiotherapy. In all but two publications the radiation target was the macroscopic recurrence. SBRT was overall well tolerated with acceptable rates of acute and late gastrointestinal or genitourinary toxicity. Quality of life was published for two phase I trials with good results. There was a very heterogeneous reporting on biochemical control after SBRT. (4) Conclusions: At this point, ultra-hypofractionated RT using SBRT to the prostate bed remains experimental and its use should be restricted to clinical trials. Given the biological rationale for extreme hypofractionation in patients with prostate cancer and the acceptable toxicity rates that have been reported, further exploration of this field is warranted.

## 1. Introduction

In patients with prostate cancer, both radical prostatectomy (RP) and radiation therapy (RT) are possible local treatment options in case of localized disease [1]. 

After any local therapy, 30–60% of patients will develop recurrent disease [2,3]. Several large randomized controlled trials have shown a benefit of adjuvant RT in patients with a high risk of local recurrence after RP, e.g., pT3 disease or positive resection margins [4,5,6,7,8]. In the era of high sensitivity prostate-specific antigen (PSA) and prostate-specific membrane antigen (PSMA) positron emission tomography-computed tomography (PET/CT), there has been additional evidence suggesting a similar oncological outcome if patients are treated with early salvage RT in case of a rising PSA after RP instead of adjuvant RT [9,10,11,12]. However, the above-mentioned studies as well as the studies including patients receiving salvage RT in case of a macroscopic tumor recurrence in the prostate bed were done with conventionally fractionated RT, usually in 2 Gy per fraction [4,5,6,7,8,9,10,11,12].

In the setting of curative treatment for localized prostate cancer, use of ultra-hypofractionated RT delivered by stereotactic body radiotherapy (SBRT) has been established as a treatment option in patients with low- or intermediate-risk. There is published data with a reasonable follow-up (FU) showing excellent biochemical control with low rates of high-grade toxicity [13,14,15,16,17,18,19,20]. In addition, data on SBRT in high-risk patients are emerging with several large trials showing encouraging results [18,21,22,23,24,25,26]. 

The rationale for using SBRT in patients with prostate cancer is the low α/β value of about 1.5 Gy [27,28]. The organs at risk in close proximity to the prostate like the bladder, rectum or urethra for instance have a higher α/β value of 3–6 [29,30,31,32]. Therefore, using a larger fraction dose is expected to improve the therapeutic ratio and consequently the probability of tumor control.

However, data on ultra-hypofractionated adjuvant or salvage RT using SBRT to the prostate bed are scarce including small phase I or retrospective studies. Potential severe acute and late toxicities are of major concern applying extreme hypofractionation in this area, especially concerning the vesicourethral anastomosis (VUA).

Data on moderate hypofractionation in the setting of postoperative RT with a fraction dose of up to 3 Gy per fraction does not seem to support this concern, given the low toxicity rates that were reported in several analyses [10,33,34,35,36,37,38,39,40,41,42,43,44,45].

We, therefore, conducted this systematic review to elaborate on the question of toxicity and oncological outcome after SBRT to the prostate bed.

## 2. Materials and Methods

### 2.1. Study Search and Selection Process

This systematic review was developed using the PICO criteria (Population, Intervention, Control, Outcome) [46,47]. The population was defined as patients with prostate cancer after RP. The intervention was defined as SBRT to either the entire prostate bed or a macroscopic tumor recurrence in the prostate bed. The control was defined as historical controls from published phase II/III studies. The outcome was defined as the following: (a) rate of acute and late toxicities after SBRT and (b) biochemical control after SBRT.

This analysis was done in accordance with the Preferred Reporting Items for Systematic Reviews and Meta-Analyses (PRISMA) recommendations [48]. This study was registered in the International Prospective Register (Research Registry; registration number reviewregistry1285). We searched the PubMed and Embase databases with the following full-text queries in August 2021: “SBRT” AND “prostate” AND “adjuvant”, “SBRT” AND “prostate” AND “postoperative”, “SBRT” AND “prostate” AND “salvage”, “stereotactic radiotherapy” AND “prostate” AND “adjuvant”, “stereotactic radiotherapy” AND “prostate” AND “postoperative”, “stereotactic radiotherapy” AND “prostate” AND “salvage”, “stereotactic radiotherapy” AND “prostate bed”, “SBRT” AND “prostate bed”. There were no limitations regarding publication date or language. 

All initially identified records were copied to an Excel sheet (Microsoft Cooperation, Redmond, WA, USA), which was used to automatically identify and remove duplicates. Out of the initially identified records, only full text articles in English reporting primary data were included in the further process. For review articles, opinions, etc., the references were checked to identify any further records that had not been identified yet. For cross reference, also terms like “extreme hypofractionation” or “ultra-hypofractionation” were considered. As the next step, only papers reporting data on adjuvant or salvage SBRT in prostate cancer patients and/or SBRT to the prostate bed were selected. Prior irradiation was not an exclusion criterion. To identify the final papers included in this analysis, papers without independent reporting of the outcome in patients with SBRT in/to the prostate bed were excluded. The identification and selection process was done twice by two of the co-authors independently (CS and RF). A third co-author served as the final judge as to which papers were included (HT).

For the section on currently ongoing trials, a search including the above-mentioned terms was conducted on clinicaltrials.gov (last search: 7 September 2021), and currently registered studies regarding adjuvant or salvage SBRT in prostate cancer patients and/or SBRT to the prostate bed were selected.

### 2.2. Data Extraction Process

The following data were extracted from the included manuscripts: first author, year of publication, journal, study design (retrospective, retrospective analysis of a prospectively collected database, prospective), study period, number of patients included, radiation treatment technique, total radiation treatment dose, target of treatment (entire prostate bed vs. macroscopic recurrence in the prostate bed), number of patients receiving androgen deprivation therapy (ADT) at the time of SBRT, dose of previous RT, time between first RT and SBRT, median FU, rates of acute and late toxicities (according to the Radiation Therapy Oncology Group (RTOG) or Common Terminology Criteria for Adverse Events (CTCAE) classification), data on biochemical control, data on applied dose constraints to organs at risk (OAR), data on target delineation and the use of markers for SBRT. The data were extracted by two independent co-authors (CS and PW) and reviewed by a third co-author (RF).

## 3. Results

### 3.1. Selected Studies

**Figure 1:**
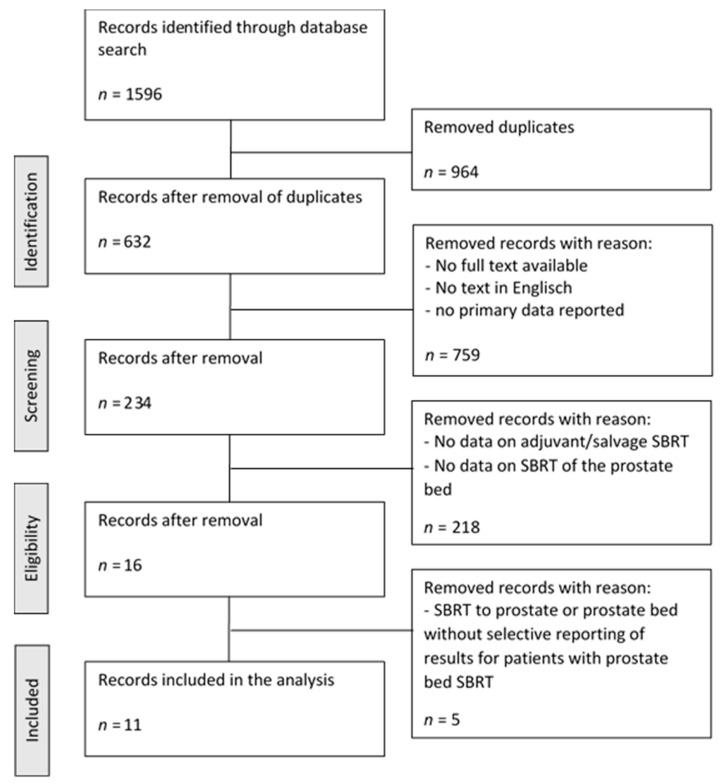
Paper selection process.

We identified a total of 1596 studies from the initial database search. From this initial set of records, 964 duplicates were removed. From the resulting 632 records, 398 records were removed due to no available full text, no record in English language or no recording of primary data. Of the remaining 234 records used for screening, 16 papers included data on patients treated with SBRT of/in the prostate bed while 218 papers were removed during this step. Of the 16 papers, 5 papers that included both patients with and without RP which did not report the results of prostate bed SBRT separately (at least toxicity or oncological outcome) were excluded, resulting in 11 publications included in this systematic review [49,50,51,52,53,54,55,56,57,58,59]. Two studies were included because of relevant information, although the patients from the respective cohorts were likely, at least in part, included in repeated reports or pooled analyses [55,59]. Figure 1 shows the consort diagram of the study selection process. Among the 11 selected papers, 2 studies were prospective phase I trials, 2 were retrospective analyses based on prospectively collected data, 1 was a case report, and 6 were retrospective analyses. Three publications included patients without prior RT, the remaining eight included patients with prior RT to the prostate or prostate bed. Table 1 and Table 2 show an overview of the included publications and further information is presented in the supplementary Table S1.

The quality of the included studies was generally low, as only two studies were prospective studies. However, these two studies were phase I studies [50,57]. The level of evidence of the included studies was consequently low with the two phase I studies being level 3 [50,57] and the remaining studies being level 4 [51,52,53,54,55,56,58,59] or level 5 [49] according to the Oxford 2011 levels of evidence [60].

### 3.2. Target Volume and Prescription Dose

Among the 11 publications, 2 were studies in which the entire prostate fossa was irradiated with SBRT in the adjuvant or salvage setting [50,57]. Both these studies were phase I trials. Ballas et al. tested three dose levels (DL) 15 × 3.6 Gy, 10 × 4.7 Gy, and 5 × 7.1 Gy in patients receiving RT on consecutive days [50]. In this study, patients after RP of any kind were included if they had pT3a/pT3b disease or T2 disease with positive surgical margins or a rising post-RP PSA level. Neoadjuvant or concurrent hormonal therapy was allowed at the discretion of the treating physician. Patients with nodal involvement and pre-irradiated patients, and patients with gross residual disease, neoadjuvant or adjuvant chemotherapy or inflammatory bowel disease were excluded. Sampath et al. did a dose escalation trial with three DL of 5 × 7 Gy, 5 × 8 Gy, and 5 × 9 Gy with patients being treated on alternate days [57]. They included patients after RP for localized prostate cancer that had either a rising PSA (up to a PSA value of 2 ng/mL), pT3a/pT3b disease or positive margins. The trial excluded node positive patients. In case of SBRT of a macroscopic recurrence in the prostate bed, patients were treated with a variety of treatment schedules. The majority of patients were treated with five to six fractions with a fraction dose of 5 to 6 Gy on alternating days [52,55,56,58]. Notably, the majority of patients receiving this fractionation schedule were re-irradiated. Patients without prior irradiation were either treated within the dose escalation phase I trials [50,57] or received 5 × 7 Gy–8 Gy (85.6% of patients in the data published by Francolini et al. [53]). 

The target volume definition differed between the studies. The gross tumor volume (GTV) was defined on the planning CT with the help of magnetic resonance imaging (MRI) or PET/CT, if applicable [49,50,52,53,54,55,56,57,58,59]. In some studies, a small clinical target volume (CTV) margin of 1–2 mm was added [53,54,55,58]. Depending on the treatment modality (e.g., Cyberknife^®^, Linac-based intensity-modulated radiotherapy (IMRT)/volumetric arc therapy (VMAT)), imaging protocol and the use of fiducial markers, an additional planning target volume (PTV) margin of 1–7 mm was added [49,50,52,53,54,55,56,57,58,59]. The details of the target delineation and the use of fiducial markers are summarized in Table S2 in the Supplementary Material.

### 3.3. Applied Dose Constraints

Of the 11 publications, details of the dose constraints used were published for 10 of them [49,50,52,53,54,55,56,57,58,59]. All ten reported one or more constraints for the rectum, either as a whole organ or for different parts (e.g., anterior rectal wall) separately. The constraints for the bladder were reported in nine studies [50,52,53,54,55,56,57,58,59]. Further, commonly used dose constraints were applied for the urethra (four studies) [49,52,53,57], femoral heads (three studies) [52,53,54], bowel (two studies) [50,53], and penile bulb (two studies) [52,53]. A summary of the reported dose constraints can be found in Table S3 in the Supplementary Material.

### 3.4. Acute and Late Toxicities

The definition of treatment related toxicity was commonly done according to the common terminology criteria for adverse events (CTCAE). The time interval for acute toxicity differed slightly between the included publications. The most common definition for acute toxicity is within 90 days/3 months. This definition with a slight range of 12 weeks to 4 months was used in five publications [50,52,56,57,58]. One publication used a cutoff of 6 months [55] and the remaining five did not specify but presumably used 3 months [49,51,53,54,59]. This slight difference in definition should be considered when interpreting the reported toxicity rates.

#### 3.4.1. Patients without Prior Radiotherapy

The overall reported rate of ≥G2 acute or late gastrointestinal (GI) and genitourinary (GU) toxicity was acceptable. Ballas et al. reported acute G2 GI toxicity in 50% of patients with only 4.2% of patients having G2 GI late toxicity [50]. The rate of acute G2 toxicity reported by Sampath et al. was lower (19.2%) but with a slightly higher rate of late G2 toxicity (11.5%) [57]. Retrospective data by Francolini et al. showed overall lower rates with 1.1% of patients having acute and late G2 toxicity, respectively [53]. No ≥ G3 GI toxicity was observed. 

Acute G2 GU toxicity was only reported by Ballas et al. (16.7%) with no patient having ≥G3 acute GU toxicity [50]. Francolini et al. reported a rate of 2.2% of patients having late G2 GU toxicity, whereas Sampath et al. even reported a rate of 38.5% late ≥ G2 toxicity, including 15.4% of patients having a late G3 toxicity [53,57]. This was a dose escalation study. When looking at the reported toxicity for the three dose levels separately, 23.1% of late ≥ G2 toxicity occurred in patients treated with 5 × 9 Gy and 13.0% in patients treated with 5 × 8 Gy. Only 2.4% (1 patient) treated with 5 × 7 Gy developed a ≥G2 late GU toxicity.

#### 3.4.2. Patients with Prior Radiotherapy

The overall rate of acute and late ≥ G2 GI toxicities was very low with only three studies reporting any G2 toxicity. Acute G2 GI toxicity was reported by Olivier et al. (8.3%) and Zerini et al. (10.0%), whereas late G2 GI toxicity was described by Jereczek-Fossa et al. (5.3%) [55,56,59]. The rate of acute GU toxicity was reasonable with only three studies describing any ≥ G2 GU toxicity in 5.3–33.3% of patients [52,55,58]. Late ≥ G2 GU toxicity was described in only three studies with a range of 11.1–26.3% [55,56,58]. No ≥ G3 acute or late GU or GI toxicities were reported. Notably, these studies had a maximum median follow up of 34.2 months.

**Figure 2:**
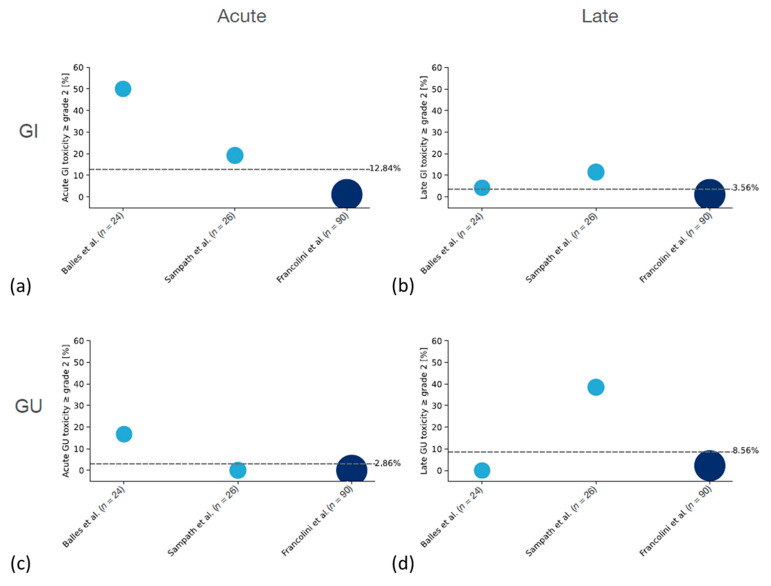
(**a**–**d**) Summary of ≥G2 acute and late toxicities in patients without prior radiotherapy. Area and color of the dots indicate the size of the respective study.

**Figure 3:**
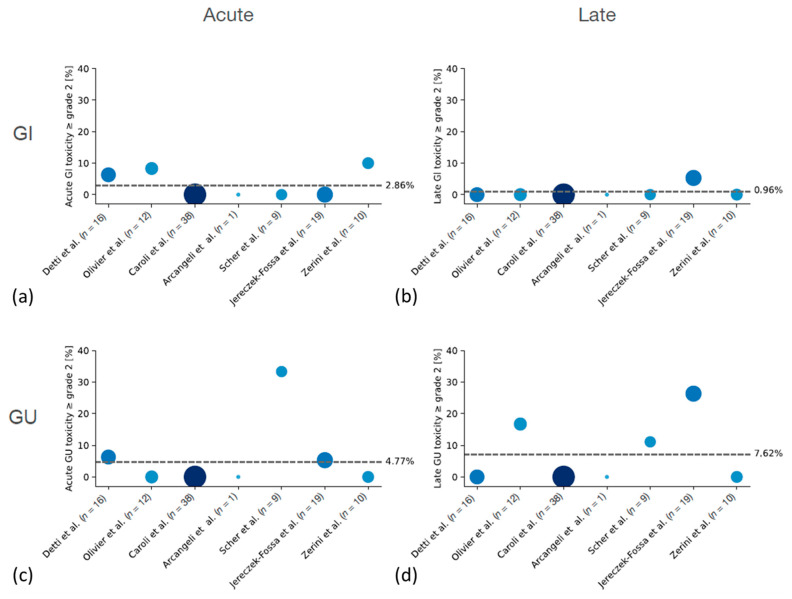
(**a**–**d**) Summary of ≥G2 acute and late toxicities in patients with prior radiotherapy. Area and color of the dots indicate the size of the respective study.

A summary of the reported rates of acute and late ≥ G2 toxicities in patients with or without prior radiotherapy is shown in Figure 2 and Figure 3.

### 3.5. Quality of Life

Quality of life was reported in the two phase I trials by Ballas et al. and Sampath et al. using common questionnaires like the International Prostate Symptom Score (IPSS), the Expanded Prostate Cancer Index Composite (EPIC-26), the Sexual Health Inventory for Men (SHIM) and the Merrick rectal function scores [50,57,61,62,63,64,65]. 

Overall IPSS was reported in both studies. Sampath et al. showed no significant change up to 24 months after treatment, Ballas et al. reported a worse IPSS in three patients from week 10 [50,57]. Generally, Sampath et al. reported very good QoL after treatment with stable values for erectile dysfunction (SHIM) and rectal QoL (Merrick rectal function score) at 24 months. Incontinence (IPSS) was worse in 14 of the initially continent patients [57]. Ballas et al. defined a minimal important difference (MID) of what difference in QoL scores is considered clinically relevant. Using the subdomains of the EPIC-26, 10 patients had worsened GI scores that met the pre-defined MID and 8 patients had incontinence scores that met MID [50].

### 3.6. Biochemical Control

Data on biochemical control was reported in ten publications, one of which did not report data on patients treated on the prostate bed separately [49,50,51,52,53,54,55,56,57,58]. 

Only two papers reported the 1-year and 2-year biochemical recurrence-free survival (bRFS) rates after SBRT, both for macroscopic recurrences after RP and external beam radiotherapy (EBRT). Olivier et al. reported 1-year and 2-year bRFS of 79 and 56% (12 patients included), respectively [56]. Janoray et al. reported a similar 1-year bRFS of 80% (10 patients) [54]. A median bRFS was only reported in two studies, 24.3 months by Francolini et al. in patients without prior radiotherapy and 15 months by Caroli et al. in patients with prior radiotherapy [51,53]. 

Biochemical response rates were reported in five studies including patients with or without prior radiotherapy with varying definition of “complete response” and time of reporting [51,52,53,57,58]. Four studies defined complete response as PSA below 0.2 ng/mL and one study as a >50% PSA reduction. Table 3 shows a summary of the complete response rates.

Patterns of failure were reported in six studies with a different approach regarding the reporting of in-field/local or out-of-field/distant failure [52,53,54,55,56,58]. Table 4 shows an overview of the reported patterns of failure.

Nine of the eleven publications included patients who received ADT at the time of SBRT treatment. However, given the small number of patients, the large heterogeneity and the inconsistent reporting of clinical data and outcome, a meaningful description of the impact of ADT is not possible.

### 3.7. Outlook on Currently Active Studies

A search of clinicaltrials.gov (accessed on 7 September 2021) as described in Section 2.1 revealed six studies including prostate bed SBRT that are currently registered. Among those, four studies are active and recruiting patients. In most of these studies, the primary endpoint is toxicity. Other primary endpoints include the maximum tolerated dose, feasibility and bRFS. All of these studies are phase I–II studies with a planned accrual of 28–102 patients. Five of these studies only include patients without prior RT, while one study includes previously irradiated patients. An overview of the currently active studies can be found in Table S4 in the Supplementary Material.

## 4. Discussion

A variety of prescription doses, target delineation concepts and RT techniques were used in the different analyses. Only in two studies, SBRT was given to the entire prostate bed. In all other studies, only the macroscopic recurrence in the prostate fossa was irradiated. Moreover, the prescribed treatment doses also differed between the studies, although the majority of patients was treated with 30–35 Gy in five to six fractions [49,50,52,53,55,56,57,58]. 

A major concern regarding SBRT after RP is radiation-induced toxicity. Even normofractionated adjuvant RT after RP is associated with significant rates of acute and late toxicities [4,5,8,66]. In the EORTC 22911 study, for instance, the highest individual acute toxicity rates of 17.3% and 17.7% G2 GU and GI toxicity were reported with ≥G3 GU and GI toxicity rates of 3.3% and 5.3% [4,5]. For late ≥ G2 GU toxicity, rates from 5% to 21.3% were reported [4,5,8,66]. The reported range of late ≥ G2 GI toxicity was generally lower in the range of 1–2.5% [4,5,8]. For patients treated with early salvage RT, the GI and GU toxicity rates were significantly lower than for adjuvant RT [9,10,11]. Sargos et al. reported late GI and GU G1-2 toxicity rates of 41% and 67% for immediate adjuvant RT and 20% and 28% for early salvage RT [11]. 

Data on adjuvant or salvage RT to the prostate bed using moderate hypofractionation showed similar toxicity rates with acute ≥ G2 GI toxicity rates ranging from 0–32.6% and ≥G2 GU toxicity rates ranging from 0–36% [33,36,37,38,40,41,42]. For late ≥ G2 GI and GU toxicity those values were 0–8% and 6.6–15% [33,36,40]. 

The impact of moderate hypofractionation in direct comparison to normofractionation in the case of adjuvant or salvage RT remains unclear. Cozzani et al. published retrospective data of patients treated in either the adjuvant or salvage setting showing an increase in acute and late GU toxicity for moderate hypofractionation [34]. However, Massaccesi et al. published data from a phase II trial including patients being treated in a similar setting showing similar rates of GI toxicity and an increased rate of G2 GU toxicity for the patients in the conventionally fractionated group [42]. Toxicity and quality of life data from the randomized, phase III NRG GU003 trial was recently published as an abstract showing non-inferiority on moderate hypofractionated radiotherapy (62.5 Gy with 2.5 Gy/fraction) compared to normofractionated radiotherapy (66.6 Gy with 1.8 Gy/fraction) with regards to late patient reported GU or GI toxicity [44]. The full publication of this data is eagerly awaited.

The rates of acute and late toxicities after prostate bed SBRT reported in the publications included in this systematic review were generally within the above-mentioned ranges. The rate of acute ≥ G2 GI and GU toxicities range 0–50% and 0–33.3% and for late ≥ G2 GI and GU toxicities range 0–11.5% and 0–38.5%, respectively [49,50,51,52,53,55,56,57,58,59]. The highest ≥ G2 GU toxicity rate of 38.5% was reported in the phase I dose escalation trial by Sampah et al. with the majority of ≥G2 GU toxicity (36.1%) occurring in the 5 × 8 and 5 × 9 Gy treatment arms [57]. 

There is not enough conclusive data to do a comprehensive comparison of patients treated with or without prior radiotherapy separately. Only three studies reported data of patients treated without prior radiotherapy, two of which treated the whole prostate bed and one treated the macroscopic recurrence only. The median reported toxicity in patients treated without prior radiotherapy seems higher when looking at the Figure 2 and Figure 3, which might be partially explained by the treatment of the whole prostate bed in the studies by Ballas et al. and Sampath et al. [50,57]. However, a factor to consider in this context is the prospective nature of these studies as compared to the others, which are retrospective analyses. Both prospective studies had a comprehensive follow up schedule which reduced the risk of underreporting of toxicity. 

Additionally, these studies were the only ones reporting quality of life. They showed an overall good outcome regarding IPSS and sexual function. A decline in GU QoL with regards to incontinence was seen in both trials [50,57]. Sampath et al. also reported unchanged rectal function at 24 months [57]. However, Ballas et al. saw a decline of the EPIC GI scores that met the threshold for MID in more than 40% of patients. This data was assessed 10 weeks after treatment, so further changes can be expected with longer follow-up [50]. 

Regarding the oncological outcome after SBRT, a comprehensive comparison of the biochemical outcome remains difficult, due to the overall short median follow-up of the included studies on prostate bed SBRT (range 6 months–40 months). Still, the reported data seems encouraging for further studies on this topic. 

Eight studies analyzed in this systematic review included patients who had undergone extensive treatment with RP, RT and ADT, if applicable. Therefore, it is not surprising that these patients showed a worse biochemical outcome after SBRT to a macroscopic recurrence. Corresponding high rates of distant failure were reported in these studies, ranging from 11.1% to 44% [52,53,56,58]. 

Oncological outcome data in patients without prior radiotherapy was published by Sampath et al. and Francolini et al. [53,57]. Using a PSA cutoff of <0.2 ng/mL, both reported biochemical control rates of around 40% with a median bRFS of 24.3 months reported by Francolini et al., which seems rather disappointing in comparison to other data on salvage RT only with a 5-year bRFS of around 50% [3]. Randomized trials including patients in the adjuvant or early salvage setting and using a normofractionated treatment regimen report even better 5-year bRFS rates of 72–89% [8,9,10]. However, the patients included in the analysis by Francolini et al. had macroscopic recurrence and a median PSA before salvage treatment of 2.3 ng/mL, which is very high compared to other studies with a median pre RT PSA of 0.2–0.8 ng/mL [67,68,69,70,71,72,73,74,75,76,77,78,79,80,81,82,83]. The pre-RT PSA levels are a known predictor of response to salvage RT [67,68,69,70,71,72,73,74,75,76,77,78,79,80,81,82,83]. A systematic review by King suggests an average loss of 2.6% with regard to relapse-free survival for each incremental 0.1 ng/mL PSA at the time of salvage RT [84]. 

For patients with prior irradiation, biochemical control rates that were reported in three analysis were even lower with a maximum of 25% and a median bRFS of 15 months [51,52,58]. These patients who had undergone extensive treatment with RP, RT and if applicable were likely at high risk of locoregional and distant recurrence with up to 44% of patients having distant recurrence [52,54,55,56]. Therefore, focal treatment as an effort to balance efficacy and possible treatment induced side effects might have been a viable treatment option in these patients to achieve local control and improve progression free survival. Due to the lack of data, a possible advantage of whole prostate bed SBRT in this setting remains unclear. Moreover, given the low number of patients included in the individual studies is low, a comprehensive analysis of the impact of ADT is not possible. 

Conclusive data regarding the oncological outcome of SBRT to the prostate fossa as well as a comparison of SBRT to the prostate fossa and normofractionated or mildly hypofractionated EBRT are missing to this date. Recruiting or active studies that were identified mainly focus on treatment induced toxicity as the primary endpoint with bRFS being a secondary endpoint in some of them. Further, data on the optimal dose for SBRT remains unclear. In the setting of re-irradiation, a dose of 5 × 6 Gy or 6 × 6 Gy is commonly used, similar to re-irradiation of the prostate [85,86,87]. This treatment dose was likely chosen in the context of the treatment dose of the first RT course, the interval between both treatments and the cumulative dose to the organs at risk. In patients without prior radiotherapy but with macroscopic recurrence, 85.6% of patients received a dose of 5 × 7–8 Gy, similar to a definite SBRT to the prostate [15,17,18,19]. Depending on the size of the macroscopic recurrence this is likely a sensible option, although definitive data are lacking. Moreover, the optimal dose to the prostate bed remains unknown. In case of normofractionated radiotherapy, the SAKK 09/10 failed to show a benefit of dose escalated radiotherapy to the prostate bed [88]. The dose of 64 Gy as given in the SAKK study would correspond to a fractionation scheme of 5 × 6 Gy with an estimated α/β value of 1.5 Gy. Whether this translates to the optimal dose scheme for stereotactic radiotherapy of the prostate bed remains unanswered by the current body of literature. However, the question remains whether a dose escalation to 5 × 8–9 Gy as done by Sampath et al. would be beneficial for better tumor control and still favorable side effect profile.

There are several limitations of this systematic review. First of all, it is limited by the small number of available studies as well as the heterogeneity of the reported data within these studies. Another major shortcoming is that we analyze and compare retrospective and prospective studies as well as studies including patients with or without a macroscopic recurrence. Unfortunately, due to the lack of sufficient prospective data, this was unavoidable. 

Due to the mostly retrospective nature of the included studies, there is certainly a risk of bias due to underreporting of events. In the context of this systematic review, the largest risk is likely the underreporting of treatment-related toxicity. With nine out of eleven included papers being of retrospective nature, this factor should be considered. Additionally, the definition of acute toxicity differed slightly between the studies. Therefore, due to the possibility of a bias, the data presentation was limited to a largely descriptive fashion. Another shortcoming of this systematic review is the short median follow up of the included studies and the lack of meaningful reporting of the influence of ADT, which could not be realized in a meaningful way due to the poor reporting and heterogeneity of the data. 

Overall, there is a lack of high-quality data on the subject of SBRT after RP to date. Therefore, the conclusions to be drawn from this systematic review are somewhat limited. However, the overall outcomes regarding toxicity and efficacy seem promising. 

## 5. Conclusions

At this point, ultra-hypofractionated RT using SBRT to the prostate bed remains experimental and its use should be restricted to clinical trials. Given the biological rationale for extreme hypofractionation in patients with prostate cancer and the acceptable toxicity rates that have been reported, further exploration of this field is warranted.

## Figures and Tables

**Table 1:** Overview of trials including patients with salvage or adjuvant prostate bed SBRT without prior radiotherapy (*n* = 3).

Trial	Year of Publication	Years of RT	Target	Number of Patients Included	Type of Trial	RT Technique	Radiotherapy Dose	Median FU	Dose Previous RT	Time between RT
Ballas et al. [50]	2019	2015–2018	Prostate bed	24	Prospective (Phase 1)	IMRT or VMAT	15 × 3.6 Gy	14.1 months	n.a.	n.a.
							10 × 4.7 Gy			
							5 × 7.1 Gy			
							Consecutive days			
Sampath et al. [57]	2020	2013–2017	Prostate bed	26	Prospective (Phase 1)	VMAT	5 × 7 Gy	40 months	n.a.	n.a.
							5 × 8 Gy			
							5 × 9 Gy			
							Alternating days			
Francolini et al. [53]	2020	2013–2018	Macroscopic recurrence	90	Retrospective	Cyber-knife or IMRT	5 × 6 Gy–9 Gy (78% 5 × 7 Gy)	21.1 months (mean)	n.a.	n.a.

**Table 2:** Overview of trials including patients with salvage prostate or prostate bed SBRT with prior radiotherapy (data below for included patients with prostate bed RT) (*n* = 8).

Trial	Year of Publication	Years of RT	Target	Number of Patients Included	Type of Trial	RT Technique	Radiotherapy Dose	Median FU	Dose Previous RT (Median)	Time between RT
Detti et al. [52]	2016	2011–2013	Macroscopic recurrence	16	retrospective analysis of prospectively collected data	Cyberknife	5 × 6 Gy (@80% IDL) for re-RT	10 months	Median 66 Gy (range 64 Gy–70 Gy)	mean 9.6 years (2.9–20.4 years)
							5 × 7 Gy (@80% IDL) for RT-naïve			
							Alternating days			
Olivier et al. [56]	2019	2011–2017	Macroscopic recurrence	12	retrospective	Cyberknife	6 × 6 Gy (@80% IDL)	34.2 months	66 Gy (11 pts)	median 77.6 months (range 21.4–160.8 months)
							Alternating days		72 Gy (1 pt)	
Caroli et al. [51]	2020	2016–2018	Not reported	38	retrospective analysis of prospectively collected data	not reported	3 × 6 Gy	27 months	not reported	not reported
							(IQR 18–21 Gy)			
Arcangeli et al. [49]	2015	not reported presumed 2014	Macroscopic recurrence	1	case report	Tomotherapy	5 × 6 Gy	6 months	66 Gy	Appr. 4 years
							Consecutive days			
Scher et al. [58]	2019	2014–2017	Macroscopic recurrence	9 (42, 21.4%)	retrospective	Cyberknife	6 × 6 Gy Alternating days	17 months	68 Gy (65–70 Gy)	128 months (54–207 months)
Jereczek-Fossa et al. [55]	2018	2009–2016	Macroscopic recurrence	19 (64, 29.7%)	retrospective	Cyberknife, Rapid Arc, Vero	5 × 5–6 Gy (median 25 Gy/5 Fx)	26.1 months	70 Gy (45–77.4 Gy)	93.9 months (27.9–183.3 months)
							Alternating days			
Zerini et al. [59]	2015	2008–2013	Macroscopic recurrence	10 (32, 31.3%)	retrospective	3D CRT (conformal dynamic arc), RA, Vero, Cyberknife	15–25 Gy/3–5 Fx	21.3 months (all patients)	73 Gy (60–83 Gy) all patients	not reported
							(median 25 Gy/5 Fx)			
							Alternating days (?)			
Janoray et al. [54]	2016	2011–2014	Macroscopic recurrence	10 (21, 48%)	retrospective	Cyberknife	5 × 7.25 Gy (@80% IDL) Alternating or consecutive days	11.7 months (all patients)	median 70 Gy (45–76 Gy)	98.03 months

**Table 3:** Summary of the complete response rates.

Study	Complete Response Defined as PSA < 0.2 ng/mL	Complete Response Defined as >50% PSA Reduction
Studies including patients without prior radiotherapy		
Sampath et al. [57]	42% (overall)	
Francolini et al. [53]	43.3% (overall after SBRT) 40% (at last follow-up)	
Studies including patients with prior radiotherapy		
Caroli et al. [51]	16.3% (at 6 months)	
Detti et al. [52]	25% (overall)	
Scher et al. [58]		83% (at last follow-up)

**Table 4:** Overview of the reported patterns of failure.

Study	In Field/Local	Locoregional/Distant	Biochemical Only	In Flied/Local and out of Field
Studies including patients without prior radiotherapy				
Francolini [53]	2.2% (local)	12.2% (locoregional and distant)	13.3%	
Studies including patients with prior radiotherapy				
Detti [52]	0	44% (distant)		
Scher [58]		11.1% (lymph nodes)	11.1%	
Jereczek-Fossa [55]	36% (in field)	21% (distant)	14%	21%
		7% (locoregional and distant)		
Janoray [54]		10% (locoregional and distant)		
Olivier [56]	16.7% local (1 outfield, 1 margin)	16.7% (distant)		16.7% (locally outfield)

## Supplementary Materials

The following supporting information can be downloaded at: https://www.mdpi.com/article/10.3390/cancers14030696/s1, Table S1: Additional Study information; Table S2: Target volume delineation and use of markers for tracking; Table S3: Dose constraints used in the individual studies; Table S4: Outlook on currently recruiting studies

## References

[1] Hamdy F.C., Donovan J.L., Lane J.A., Mason M., Metcalfe C., Holding P., Davis M., Peters T.J., Turner E.L., Martin R.M. (2016). 10-Year Outcomes after Monitoring, Surgery, or Radiotherapy for Localized Prostate Cancer. N. Engl. J. Med..

[2] Han M., Partin A.W., Zahurak M., Piantadosi S., Epstein I.J., Walsh P.C. (2003). Biochemical (prostate specific antigen) recurrence probability following radical prostatectomy for clinically localized prostate cancer. J. Urol..

[3] Gandaglia G., Briganti A., Clarke N., Karnes R.J., Graefen M., Ost P., Zietman A.L., Roach M. (2017). Adjuvant and Salvage Radiotherapy after Radical Prostatectomy in Prostate Cancer Patients. Eur. Urol..

[4] Bolla M., van Poppel H., Collette L., van Cangh P., Vekemans K., Da Pozzo L.F., de Reijke T.M., Verbaeys A., Bosset J.-F., van Velthoven R. (2005). Postoperative radiotherapy after radical prostatectomy: A randomised controlled trial (EORTC trial 22911). Lancet.

[5] Bolla M., van Poppel H., Tombal B., Vekemans K., Da Pozzo L.F., de Reijke T.M., Verbaeys A., Bosset J.-F., van Velthoven R., Colombel M. (2012). Postoperative radiotherapy after radical prostatectomy for high-risk prostate cancer: Long-term results of a randomised controlled trial (EORTC trial 22911). Lancet.

[6] Thompson I.M., Tangen C.M., Paradelo J., Lucia M.S., Miller G., Troyer D., Messing E., Forman J., Chin J., Swanson G. (2006). Adjuvant Radiotherapy for Pathologically Advanced Prostate Cancer: A Randomized Clinical Trial. JAMA.

[7] Thompson I.M., Tangen C.M., Paradelo J., Lucia M.S., Miller G., Troyer D., Messing E., Forman J., Chin J., Swanson G. (2009). Adjuvant Radiotherapy for Pathological T3N0M0 Prostate Cancer Significantly Reduces Risk of Metastases and Improves Survival: Long-Term Followup of a Randomized Clinical Trial. J. Urol..

[8] Wiegel T., Bottke D., Steiner U., Siegmann A., Golz R., Störkel S., Willich N., Semjonow A., Souchon R., Stöckle M. (2009). Phase III Postoperative Adjuvant Radiotherapy After Radical Prostatectomy Compared with Radical Prostatectomy Alone in pT3 Prostate Cancer with Postoperative Undetectable Prostate-Specific Antigen: ARO 96-02/AUO AP 09/95. J. Clin. Oncol..

[9] Kneebone A., Fraser-Browne C., Duchesne G.M., Fisher R., Frydenberg M., Herschtal A., Williams S.G., Brown C., Delprado W., Haworth A. (2020). Adjuvant radiotherapy versus early salvage radiotherapy following radical prostatectomy (TROG 08.03/ANZUP RAVES): A randomised, controlled, phase 3, non-inferiority trial. Lancet Oncol..

[10] Parker C.C., Clarke N.W., Cook A.D., Kynaston H.G., Petersen P.M., Catton C., Cross W., Logue J., Parulekar W., Payne H. (2020). Timing of radiotherapy after radical prostatectomy (RADICALS-RT): A randomised, controlled phase 3 trial. Lancet.

[11] Sargos P., Chabaud S., Latorzeff I., Magné N., Benyoucef A., Supiot S., Pasquier D., Abdiche M.S., Gilliot O., Graff-Cailleaud P. (2020). Adjuvant radiotherapy versus early salvage radiotherapy plus short-term androgen deprivation therapy in men with localised prostate cancer after radical prostatectomy (GETUG-AFU 17): A randomised, phase 3 trial. Lancet Oncol..

[12] Vale C.L., Fisher D., Kneebone A., Parker C., Pearse M., Richaud P., Sargos P., Sydes M.R., Brawley C., Brihoum M. (2020). Adjuvant or early salvage radiotherapy for the treatment of localised and locally advanced prostate cancer: A prospectively planned systematic review and meta-analysis of aggregate data. Lancet.

[13] Boike T.P., Lotan Y., Cho L.C., Brindle J., Derose P., Xie X.-J., Yan J., Foster R., Pistenmaa D., Perkins A. (2011). Phase I Dose-Escalation Study of Stereotactic Body Radiation Therapy for Low- and Intermediate-Risk Prostate Cancer. J. Clin. Oncol..

[14] Brand D.H., Tree A.C., Ostler P., Van Der Voet H., Loblaw A., Chu W., Ford D., Tolan S., Jain S., Martin A. (2019). Intensity-modulated fractionated radiotherapy versus stereotactic body radiotherapy for prostate cancer (PACE-B): Acute toxicity findings from an international, randomised, open-label, phase 3, non-inferiority trial. Lancet Oncol..

[15] Chen L.N., Suy S., Uhm S., Oermann E.K., Ju A.W., Chen V., Hanscom H.N., Laing S., Kim J.S., Lei S. (2013). Stereotactic Body Radiation Therapy (SBRT) for clinically localized prostate cancer: The Georgetown University experience. Radiat. Oncol..

[16] Jackson W.C., Silva J., Hartman H.E., Dess R.T., Kishan A.U., Beeler W.H., Gharzai L.A., Jaworski E.M., Mehra R., Hearn J.W.D. (2019). Stereotactic body radiation therapy for localized prostate cancer: A systematic review and meta-analysis of over 6,000 patients treated on prospective studies. Int. J. Radiat. Oncol. Biol. Phys..

[17] King C.R., Brooks J.D., Gill H., Presti J.C. (2012). Long-Term Outcomes from a Prospective Trial of Stereotactic Body Radiotherapy for Low-Risk Prostate Cancer. Int. J. Radiat. Oncol..

[18] Kotecha R., Djemil T., Tendulkar R.D., Reddy C., Thousand R.A., Vassil A., Stovsky M., Berglund R.K., Klein E.A., Stephans K.L. (2016). Dose-Escalated Stereotactic Body Radiation Therapy for Patients with Intermediate- and High-Risk Prostate Cancer: Initial Dosimetry Analysis and Patient Outcomes. Int. J. Radiat. Oncol..

[19] Meier R.M., Bloch D.A., Cotrutz C., Beckman A.C., Henning G.T., Woodhouse S.A., Williamson S.K., Mohideen N., Dombrowski J.J., Hong R.L. (2018). Multicenter Trial of Stereotactic Body Radiation Therapy for Low- and Intermediate-Risk Prostate Cancer: Survival and Toxicity Endpoints. Int. J. Radiat. Oncol..

[20] Zilli T., Franzese C., Bottero M., Giaj-Levra N., Foerster R., Zwahlen D., Koutsouvelis N., Bertaut A., Blanc J., D’Agostino G.R. (2019). Single fraction urethra-sparing prostate cancer SBRT: Phase I results of the ONE SHOT trial. Radiother. Oncol..

[21] Bauman G., Ferguson M., Lock M., Chen J., Ahmad B., Venkatesan V., Sexton T., D’Souza D., Loblaw A., Warner A. (2015). A Phase 1/2 Trial of Brief Androgen Suppression and Stereotactic Radiation Therapy (FASTR) for High-Risk Prostate Cancer. Int. J. Radiat. Oncol..

[22] Callan L., Bauman G., Chen J., Lock M., Sexton T., D’Souza D., Rodrigues G. (2019). A Phase I/II Trial of Fairly Brief Androgen Suppression and Stereotactic Radiation Therapy for High-Risk Prostate Cancer (FASTR-2): Preliminary Results and Toxicity Analysis. Adv. Radiat. Oncol..

[23] Foerster R., Zwahlen D., Buchali A., Tang H., Schroeder C., Windisch P., Vu E., Akbaba S., Bostel T., Sprave T. (2021). Stereotactic Body Radiotherapy for High-Risk Prostate Cancer: A Systematic Review. Cancers.

[24] Murthy V., Gupta M., Mulye G., Maulik S., Munshi M., Krishnatry R., Phurailatpam R., Mhatre R., Prakash G., Bakshi G. (2018). Early Results of Extreme Hypofractionation Using Stereotactic Body Radiation Therapy for High-risk, Very High-risk and Node-positive Prostate Cancer. Clin. Oncol..

[25] Widmark A., Gunnlaugsson A., Beckman L., Thellenberg-Karlsson C., Hoyer M., Lagerlund M., Kindblom J., Ginman C., Johansson B., Björnlinger K. (2019). Ultra-hypofractionated versus conventionally fractionated radiotherapy for prostate cancer: 5-year outcomes of the HYPO-RT-PC randomised, non-inferiority, phase 3 trial. Lancet.

[26] Zilli T., Jorcano S., Bral S., Rubio C., Bruynzeel A.M., Oliveira A., Abacioglu U., Minn H., Symon Z., Miralbell R. (2020). Once-a-week or every-other-day urethra-sparing prostate cancer stereotactic body radiotherapy, a randomized phase II trial: 18 months follow-up results. Cancer Med..

[27] Brenner D.J., Martinez A.A., Edmundson G.K., Mitchell C., Thames H.D., Armour E.P. (2002). Direct evidence that prostate tumors show high sensitivity to fractionation (low α/β ratio), similar to late-responding normal tissue. Int. J. Radiat. Oncol..

[28] Miralbell R., Roberts S., Zubizarreta E., Hendry J.H. (2012). Dose-Fractionation Sensitivity of Prostate Cancer Deduced from Radiotherapy Outcomes of 5,969 Patients in Seven International Institutional Datasets: α/β = 1.4 (0.9–2.2) Gy. Int. J. Radiat. Oncol..

[29] Wang K., Mavroidis P., Royce T.J., Falchook A.D., Collins S.P., Sapareto S., Sheets N.C., Fuller D.B., El Naqa I., Yorke E. (2021). Prostate Stereotactic Body Radiation Therapy: An Overview of Toxicity and Dose Response. Int. J. Radiat. Oncol..

[30] Gloi A.M., Buchanan R. (2013). Dosimetric assessment of prostate cancer patients through principal component analysis (PCA). J. Appl. Clin. Med. Phys..

[31] Daşu A. (2007). Is the α/β Value for Prostate Tumours Low Enough to be Safely Used in Clinical Trials?. Clin. Oncol..

[32] Brenner D.J. (2004). Fractionation and late rectal toxicity. Int. J. Radiat. Oncol..

[33] Barra S., Belgioia L., Marcenaro M., Callegari S., Pastorino A., Trapani L., Cavagnetto F., Garelli S., Corvo R. (2018). Moderate hypofractionated radiotherapy after prostatectomy for cancer patients: Toxicity and clinical outcome. Cancer Manag. Res..

[34] Cozzarini C., Fiorino C., Deantoni C., Briganti A., Fodor A., La Macchia M., Chiorda B.N., Rancoita P.M.V., Suardi N., Zerbetto F. (2014). Higher-than-expected Severe (Grade 3–4) Late Urinary Toxicity After Postprostatectomy Hypofractionated Radiotherapy: A Single-institution Analysis of 1176 Patients. Eur. Urol..

[35] Cozzarini C., Fiorino C., Di Muzio N., Valdagni R., Salonia A., Alongi F., Broggi S., Guazzoni G., Montorsi F., Rigatti P. (2008). Hypofractionated adjuvant radiotherapy with helical Tomotherapy after radical prostatectomy: Planning data and toxicity results of a Phase I–II study. Radiother. Oncol..

[36] Fersino S., Tebano U., Mazzola R., Giaj-Levra N., Ricchetti F., Di Paola G., Fiorentino A., Sicignano G., Naccarato S., Ruggieri R. (2017). Moderate Hypofractionated Postprostatectomy Volumetric Modulated Arc Therapy With Daily Image Guidance (VMAT-IGRT): A Mono-institutional Report on Feasibility and Acute Toxicity. Clin. Genitourin. Cancer.

[37] Ippolito E., Cellini N., Digesù C., Cilla S., Mantini G., Balducci M., Di Lallo A., Deodato F., Macchia G., Massaccesi M. (2013). Postoperative intensity-modulated radiotherapy with simultaneous integrated boost in prostate cancer: A dose-escalation trial. Urol. Oncol. Semin. Orig. Investig..

[38] Katayama S., Striecker T., Kessel K., Sterzing F., Habl G., Edler L., Debus J., Herfarth K. (2014). Hypofractionated IMRT of the Prostate Bed After Radical Prostatectomy: Acute Toxicity in the PRIAMOS-1 Trial. Int. J. Radiat. Oncol..

[39] Krause S., Sterzing F., Neuhof D., Edler L., Debus J., Herfarth K. (2012). Hypofractionated helical intensity-modulated radiotherapy of the prostate bed after prostatectomy with or without the pelvic lymph nodes—the PRIAMOS trial. BMC Cancer.

[40] Kruser T., Jarrard D.F., Graf A.K., Hedican S.P., Paolone D.R., Wegenke J.D., Liu G., Ms H.M.G., Ritter M.A. (2010). Early hypofractionated salvage radiotherapy for postprostatectomy biochemical recurrence. Cancer.

[41] Lewis S.L., Patel P., Song H., Freedland S.J., Bynum S., Oh D., Palta M., Yoo D., Oleson J., Salama J.K. (2016). Image Guided Hypofractionated Postprostatectomy Intensity Modulated Radiation Therapy for Prostate Cancer. Int. J. Radiat. Oncol..

[42] Massaccesi M., Cilla S., Deodato F., Digesù C., Macchia G., Caravatta L., Ippolito E., Picardi V., Ferro M., Mignogna S. (2013). Hypofractionated intensity-modulated radiotherapy with simultaneous integrated boost after radical prostatectomy: Preliminary results of a phase II trial. Anticancer. Res..

[43] Chin S., Fatimilehin A., Walshaw R., Argarwal A., Mistry H., Elliott T., Logue J., Wylie J., Choudhury A. (2020). Ten-Year Outcomes of Moderately Hypofractionated Salvage Postprostatectomy Radiation Therapy and External Validation of a Contemporary Multivariable Nomogram for Biochemical Failure. Int. J. Radiat. Oncol..

[44] Buyyounouski M., Pugh S., Chen R., Mann M., Kudchadker R., Konski A., Mian O., Michalski J., Vigneault E., Valicenti R. (2021). Primary Endpoint Analysis of a Randomized Phase III Trial of Hypofractionated vs. Conventional Post-Prostatectomy Radiotherapy: NRG Oncology GU003. Int. J. Radiat. Oncol..

[45] Picardi C., Perret I., Miralbell R., Zilli T. (2018). Hypofractionated radiotherapy for prostate cancer in the postoperative setting: What is the evidence so far?. Cancer Treat. Rev..

[46] Richardson W.S., Wilson M.C., Nishikawa J., Hayward R.S. (1995). The well-built clinical question: A key to evidence-based decisions. ACP J. Club.

[47] Sackett D.L. (1997). Evidence-based medicine. Semin. Perinatol..

[48] Page M.J., McKenzie J.E., Bossuyt P.M., Boutron I., Hoffmann T.C., Mulrow C.D., Shamseer L., Tetzlaff J.M., Akl E.A., Brennan S.E. (2021). The PRISMA 2020 statement: An updated guideline for reporting systematic reviews. BMJ.

[49] Arcangeli S., Gambardella P., Agolli L., Monaco A., Dognini J., Regine G., Donato V. (2015). Stereotactic Body Radiation Therapy Salvage Reirradiation of Radiorecurrent Prostatic Carcinoma Relapsed in the Prostatic Bed. Tumori J..

[50] Ballas L.K., Luo C., Chung E., Kishan A.U., Shuryak I., Quinn D.I., Dorff T., Jhimlee S., Chiu R., Abreu A. (2019). Phase 1 Trial of SBRT to the Prostate Fossa After Prostatectomy. Int. J. Radiat. Oncol..

[51] Caroli P., Colangione S.P., De Giorgi U., Ghigi G., Celli M., Scarpi E., Monti M., Di Iorio V., Sarnelli A., Paganelli G. (2020). ^68^Ga-PSMA-11 PET/CT-Guided Stereotactic Body Radiation Therapy Retreatment in Prostate Cancer Patients with PSA Failure after Salvage Radiotherapy. Biomedicines.

[52] Detti B., Bonomo P., Masi L., Doro R., Cipressi S., Iermano C., Bonucci I., Franceschini D., Di Brina L., Baki M. (2015). CyberKnife stereotactic radiotherapy for isolated recurrence in the prostatic bed. World J. Urol..

[53] Francolini G., Jereczek-Fossa B.A., Di Cataldo V., Simontacchi G., Marvaso G., Zerella M.A., Gentile P., Bianciardi F., Allegretta S., Detti B. (2020). Stereotactic radiotherapy for prostate bed recurrence after prostatectomy, a multicentric series. BJU Int..

[54] Janoray G., Reynaud-Bougnoux A., Ruffier-Loubière A., Bernadou G., Pointreau Y., Calais G. (2016). Stereotactic body reirradiation therapy for locally recurrent prostate cancer after external-beam radiation therapy: Initial report. Cancer Radiothérapie.

[55] Jereczek-Fossa B.A., Rojas D.P., Zerini D., Fodor C.I., Viola A., Fanetti G., Volpe S., Luraschi R., Bazani A., Rondi E. (2019). Reirradiation for isolated local recurrence of prostate cancer: Mono-institutional series of 64 patients treated with salvage stereotactic body radiotherapy (SBRT). Br. J. Radiol..

[56] Olivier J., Basson L., Puech P., Lacornerie T., Villers A., Wallet J., Lartigau E., Pasquier D. (2019). Stereotactic Re-irradiation for Local Recurrence in the Prostatic Bed After Prostatectomy: Preliminary Results. Front. Oncol..

[57] Sampath S., Frankel P., del Vecchio B., Ruel N., Yuh B., Liu A., Tsai T., Wong J. (2020). Stereotactic Body Radiation Therapy to the Prostate Bed: Results of a Phase 1 Dose-Escalation Trial. Int. J. Radiat. Oncol..

[58] Scher N., Bauduceau O., Bollet M., Lamallem H., Charas T., Garaud P., Foster D., Fawzi M., Labidi M., Toledano A. (2019). Stereotactic prostate focal reirradiation therapy for local recurrence: Preliminary results of Hartmann Oncology Radiotherapy Group. BJR Open.

[59] Zerini D., Jereczek-Fossa A.B., Fodor C.I., Bazzani F., Maucieri A., Ronchi S., Ferrario S., Colangione S.P., Gerardi A.M., Caputo M. (2015). Salvage image-guided intensity modulated or stereotactic body reirradiation of local recurrence of prostate cancer. Br. J. Radiol..

[60] Howick J., Glasziou P., Greenhalgh T., Heneghan C., Liberati A., Moschetti I., Phillips B., Thornton H., OCEBM Table of Evidence Working Group, OCEBM Levels of Evidence Working Group (2021). The Oxford 2011 Levels of Evidence.

[61] Merrick G.S., Butler W.M., Dorsey A.T., Galbreath R.W., Blatt H., Lief J.H. (2000). Rectal function following prostate brachytherapy. Int. J. Radiat. Oncol..

[62] Rosen R., Cappelleri J., Smith M.D., Lipsky J., Peña B. (1999). Development and evaluation of an abridged, 5-item version of the International Index of Erectile Function (IIEF-5) as a diagnostic tool for erectile dysfunction. Int. J. Impot. Res..

[63] Barry M.J., Fowler F.J., O’Leary M.P., Bruskewitz R.C., Holtgrewe H.L., Mebust W.K., Cockett A.T. (1992). The Measurement Committee of the American Urological Association the American Urological Association Symptom Index for Benign Prostatic Hyperplasia. The Measurement Committee of the American Urological Association. J. Urol..

[64] Badia X., Garcia-Losa M., Dal-Ré R. (1997). Ten-Language Translation and Harmonization of the International Prostate Symptom Score: Developing a Methodology for Multinational Clinical Trials. Eur. Urol..

[65] Wei J.T., Dunn R.L., Litwin M., Sandler H.M., Sanda M.G. (2000). Development and validation of the expanded prostate cancer index composite (EPIC) for comprehensive assessment of health-related quality of life in men with prostate cancer. Urology.

[66] Mak R.H., Hunt D., Efstathiou J.A., Heney N.M., Jones C.U., Lukka H.R., Bahary J.-P., Patel M., Balogh A., Nabid A. (2016). Acute and late urinary toxicity following radiation in men with an intact prostate gland or after a radical prostatectomy: A secondary analysis of RTOG 94-08 and 96-01. Urol. Oncol. Semin. Orig. Investig..

[67] Fiorino C., Broggi S., Fossati N., Cozzarini C., Goldner G., Wiegel T., Hinkelbein W., Karnes R.J., Boorjian S.A., Haustermans K. (2016). Predicting the 5-Year Risk of Biochemical Relapse After Postprostatectomy Radiation Therapy in ≥PT2, pN0 Patients with a Comprehensive Tumor Control Probability Model. Int. J. Radiat. Oncol..

[68] Tendulkar R.D., Agrawal S., Gao T., Efstathiou J.A., Pisansky T.M., Michalski J.M., Koontz B.F., Hamstra D.A., Feng F.Y., Liauw S.L. (2016). Contemporary Update of a Multi-Institutional Predictive Nomogram for Salvage Radiotherapy After Radical Prostatectomy. J. Clin. Oncol..

[69] Stish B.J., Pisansky T.M., Harmsen W.S., Davis B., Tzou K.S., Choo R., Buskirk S.J. (2016). Improved Metastasis-Free and Survival Outcomes with Early Salvage Radiotherapy in Men with Detectable Prostate-Specific Antigen after Prostatectomy for Prostate Cancer. J. Clin. Oncol..

[70] Fossati N., Karnes R.J., Cozzarini C., Fiorino C., Gandaglia G., Joniau S., Boorjian S.A., Goldner G., Hinkelbein W., Haustermans K. (2016). Assessing the Optimal Timing for Early Salvage Radiation Therapy in Patients with Prostate-specific Antigen Rise After Radical Prostatectomy. Eur. Urol..

[71] Briganti A., Karnes R.J., Joniau S., Boorjian S.A., Cozzarini C., Gandaglia G., Hinkelbein W., Haustermans K., Tombal B., Shariat S. (2014). Prediction of Outcome Following Early Salvage Radiotherapy Among Patients with Biochemical Recurrence After Radical Prostatectomy. Eur. Urol..

[72] Van Der Poel H.G., Tillier C., De Blok W., Acar C., Van Muilekom E.H. (2013). Salvage Radiotherapy After Robot-assisted Laparoscopic Radical Prostatectomy. Urology.

[73] Song W., Jeon H.G., Sung H.H., Jeong B.C., Seo S.I., Jeon S.S., Choi H.Y., Lee H.M. (2015). Prognostic factors after salvage radiotherapy alone in patients with biochemical recurrence after radical prostatectomy. Int. J. Urol..

[74] Ploussard G., Staerman F., Pierrevelcin J., LaRue S., Villers A., Ouzzane A., Bastide C., Gaschignard N., Buge F., The Committee of Cancerology (CCAFU) of the Association of French Urology (AFU) (2014). Clinical outcomes after salvage radiotherapy without androgen deprivation therapy in patients with persistently detectable PSA after radical prostatectomy: Results from a national multicentre study. World J. Urol..

[75] Parekh A., Chen M.-H., Graham P., Mahal B.A., Hirsch A.E., Nakabayashi M., Evan C., Kantoff P., Martin N.E., Nguyen P.L. (2015). Role of Androgen Deprivation Therapy in Early Salvage Radiation Among Patients with Prostate-Specific Antigen Level of 0.5 or Less. Clin. Genitourin. Cancer.

[76] Lohm G., Lütcke J., Jamil B., Höcht S., Neumann K., Hinkelbein W., Wiegel T., Bottke D. (2014). Salvage radiotherapy in patients with prostate cancer and biochemical relapse after radical prostatectomy: Long-Term Follow-Up of A Single-Center Survey. Strahlenther. und Onkol..

[77] Kwon O., Kim K.B., Lee Y.I., Byun S.-S., Kim J.-S., Lee S.E., Hong S.K. (2014). Salvage Radiotherapy after Radical Prostatectomy: Prediction of Biochemical Outcomes. PLoS ONE.

[78] Ervandian M., Høyer M., Petersen S.E., Sengeløv L., Hansen S., Holmberg M., Sveistrup J., Petersen P.M., Borre M. (2015). Salvage radiation therapy following radical prostatectomy. A national Danish study. Acta Oncol..

[79] Blanchard P., Bakkour M., De Crevoisier R., Levy A., Baumert H., Patard J.-J., Wibault P., Fizazi K., Bossi A. (2015). Early PSA level decline is an independent predictor of biochemical and clinical control for salvage postprostatectomy radiotherapy. Urol. Oncol. Semin. Orig. Investig..

[80] Moreira D.M., Jayachandran J., Presti J.C., Aronson W.J., Terris M.K., Kane C.J., Amling C.L., Stephenson A.J., Freedland S.J. (2009). Validation of a nomogram to predict disease progression following salvage radiotherapy after radical prostatectomy: Results from the SEARCH database. Br. J. Urol..

[81] Bs W.J., Hamstra D.A., Bs S.J., Zhou J., Bs B.F., Foster C., Li D., Song Y., Palapattu G.S., Kunju L.P. (2013). Gleason pattern 5 is the strongest pathologic predictor of recurrence, metastasis, and prostate cancer-specific death in patients receiving salvage radiation therapy following radical prostatectomy. Cancer.

[82] Goenka A., Magsanoc J.M., Pei X., Schechter M., Kollmeier M., Cox B., Scardino P.T., Eastham J.A., Zelefsky M.J. (2012). Long-Term Outcomes After High-Dose Postprostatectomy Salvage Radiation Treatment. Int. J. Radiat. Oncol..

[83] Ost P., De Troyer B., Fonteyne V., Oosterlinck W., De Meerleer G. (2011). A Matched Control Analysis of Adjuvant and Salvage High-Dose Postoperative Intensity-Modulated Radiotherapy for Prostate Cancer. Int. J. Radiat. Oncol..

[84] King C.R. (2012). The Timing of Salvage Radiotherapy after Radical Prostatectomy: A Systematic Review. Int. J. Radiat. Oncol..

[85] Pasquier D., Martinage G., Janoray G., Rojas D.P., Zerini D., Goupy F., De Crevoisier R., Bogart E., Calais G., Toledano A. (2019). Salvage Stereotactic Body Radiation Therapy for Local Prostate Cancer Recurrence after Radiation Therapy: A Retrospective Multicenter Study of the GETUG. Int. J. Radiat. Oncol..

[86] Loi M., Di Cataldo V., Simontacchi G., Detti B., Bonomo P., Masi L., Desideri I., Greto D., Francolini G., Carfora V. (2018). Robotic Stereotactic Retreatment for Biochemical Control in Previously Irradiated Patients Affected by Recurrent Prostate Cancer. Clin. Oncol..

[87] Cuccia F., Nicosia L., Mazzola R., Figlia V., Giaj-Levra N., Ricchetti F., Rigo M., Vitale C., Corradini S., Ruggieri R. (2020). Linac-based SBRT as a feasible salvage option for local recurrences in previously irradiated prostate cancer. Strahlenther. Onkol..

[88] Ghadjar P., Hayoz S., Bernhard J., Zwahlen D.R., Hölscher T., Gut P., Polat B., Hildebrandt G., Müller A.-C., Plasswilm L. (2021). Dose-intensified Versus Conventional-dose Salvage Radiotherapy for Biochemically Recurrent Prostate Cancer after Prostatectomy: The SAKK 09/10 Randomized Phase 3 Trial. Eur. Urol..

