# Supplementary material for: Stereotactic Radiotherapy after Radical Prostatectomy in Patients with Prostate Cancer in the Adjuvant or Salvage Setting: A Systematic Review
Source: Cancers (Basel). 2022 Jan 29;14(3):696. doi: 10.3390/cancers14030696 (PMC8833497; doi:10.3390/cancers14030696)
Supplement: Supplementary file 1 [file cancers-14-00696-s001.zip › cancers-1564713-supplementary.pdf]

Table S1. Additional Study information.

Trial	Inclusion Criteria	Exclusion Criteria	ADT	No. patients receiving ADT
Ballas et al. (46)	≥ 18 years<br>RPE of any kind<br>ECOG 0-1<br>T2 if R+ or high postop PSA, pT3a/pT3b	N+, M1<br>Prior RT to the pelvis<br>Gross residual disease<br>Neoadjuvant or postoperative chemotherapy<br>History of inflammatory bowel disease	At the discretion of the treating physician	4
Sampath et al. (53)	RPE for localized prostate cancer rising PSA after surgery up to a value of 2.0<br>pT3a-b disease<br>R+	N+	At the discretion of the treating physician	10
Francolini et al. (49)	previous RP, developed isolated prostate bed macroscopic recurrence	N+<br>M1	At the discretion of the treating physician	17
Detti et al. (48)	previous RP, developed isolated prostate bed macroscopic recurrence	N+<br>M1	Not specified	0
Olivier et al. (52)	previous RP, developed isolated prostate bed macroscopic recurrence	N+<br>M1	Not specified, presumably at the discretion of the treating physician	2
Caroli et al. (47)	previous RT on the prostate bed PSA failure after RT<br>positive PSMA PET/CT in the prostate bed only<br>Re-RT guided by PSMA PET/CT<br>complete clinical follow-up available	negative PSMA PET/CT in the prostate bed<br>PSMA PET/CT positive outside the bed	Not allowed	0
Arcangeli et al. (45)	previous RP,<br>Macroscopic failure in the prostate bed	n.a.	n.a.	0
Scher et al. (54)	previous RP, presence of a single recurrence from prostate cancer<br>Interval between first diagnosis of prostate cancer and diagnosis of recurrent disease greater ≥2 years<br>No severe (G≥3) chronic late toxicity	N+<br>M1<br>Another salvage local therapy	At the discretion of the treating physician, patients receiving ADT excluded from evaluation of biochemical response	3
Jereczek-Fossa et al. (51)	isolated local recurrence after primary EBRT, BRT or salvage RT (for systematic review post RPE only)<br>minimum follow-up of 3 months	metastatic disease<br>other local salvage treatment for the recurrent PCa	At the discretion of the treating physician	6
Zerini et al. (55)	isolated local recurrence of prostate cancer after radical or post-RPE RT<br>follow-up of at least 12 months	N+<br>M1<br>Another salvage local therapy	Not specified, presumably at the discretion of the treating physician	3
Janoray et al. (50)	≥ 18 years<br>single recurrence within the prostate gland, seminal vesicles, or anastomosis<br>previously treated with conventional radiotherapy	N+<br>M1	Not specified, presumably at the discretion of the treating physician	1

Table S2. Target volume delineation and use of markers for tracking .

	Target delineation	Markers
Ballas et al.	CTV according to the EORTC guideline postoperative RT (covered vesicourethral anastomosis and bladder neck, carved out of the bladder superior to that<br>PTV = CTV + 3 mm	3 fiducial markers (correspond to what would have been the right mid gland, left base, and left apex of an intact prostate gland)
Sampath et al.	CTV included the prostate fossa, distal bladder neck, and VUA<br>PTV = CTV + 2 mm	3 gold inert fiducial markers in the prostate bed
Francolini et al.	GTV corresponded to macroscopic neoplastic tissue in the prostate bed<br>CTV = GTV + 2 mm<br>PTV = CTV + 3 mm (1 mm in the posterior direction) (Cyberknife)<br>PTV = CTV + 5.5 mm (1–3.5 mm in the posterior direction) (VERO IMRT)<br>The bladder was excluded from the target volume	Fiducial markers were implanted in CyberKnife patients
Detti et al.	GTV was defined on the basis of clinical and radiological findings<br>PTV = GTV + 2 mm	Fiducial tracking
Olivier et al.	GTV was defined as macroscopic local recurrence on imaging<br>PTV = GTV + 2 mm	One internal gold fiducial was placed in contact with the lesion
Caroli et al.	not reported	not reported
Arcangeli et al.	CTV was defined as the site of recurrence<br>PTV = CTV + 5 mm	none
Scher et al.	GTV was defined as macroscopic recurrence<br>CTV = GTV + 1 mm<br>PTV = CTV + 1 mm	4 gold fiducial markers were placed in the prostate bed
Jereczek-Fossa et al.	GTV contouring was based on the mpMRI and PET/CT co-registration<br>CTV = GTV with margins<br>PTV = CTV + 5 mm in all directions (3 mm for the posterior margin)	Fiducial markers were implanted in CyberKnife patients
Zerini et al.	Cyberknife: GTV + 1-mm margin to take account of marker detection inaccuracy<br>Other: CTV included the site of relapse in the prostatic bed<br>PTV = CTV + 5 mm (3 mm posteriorly) (Cyberknife, VERO)<br>PTV = CTV + 7 mm (5 mm posteriorly) (for IG-3D-CRT)	Fiducial markers were implanted in CyberKnife patients
Janoray et al.	GTV was defined as macroscopic recurrence<br>CTV = GTV + 1 mm for low- and intermediate-risk prostate cancer, GTV + 2 mm for high-risk prostate cancer<br>PTV = CTV + 3 mm margin (1 mm posteriorly)	none (Xsight®Spine)

Table S3. Dose constraints used in the individual studies.

		Rectum	Bladder	Urethra	other
Including patients without prior RT	Ballas et al.	Anterior rectal wall: Dmax < 105% of PD<br>Lateral walls: V(90% of PD) < 3cc<br>Posterior rectal wall: Dmax ≤ 45% of PD, V24Gy < 50%<br>Rectal volume: V50 Gy < 3cc<br>Rectal circumference: V39 Gy < 35%	Bladder wall*: Dmax < 105% of PD<br>If this constraint could not be met:<br>V40 Gy < 22.9 cc, V50 Gy < 13.2 cc		Bowel: D0.5cc < 30 Gy
	Sampath et al.	Anterior rectal wall: Dmax < 105% of PD, V(PD) < 30%	Bladder wall*: Dmax < 105% of PD<br>V18.3 Gy < 20cc (25cc acceptable)	distal urethra Dmax < 105% of PD	Vesical-Urethral anastomosis: Dmax < 105% of PD
	Francolini et al.	V18.1 Gy < 50%<br>V29 Gy < 20%<br>V36 Gy < 1cc	V18.1 Gy < 40%<br>V37 Gy < 10cc	V42 Gy < 50% (not man-datory)	Femoral Heads: 14.5 Gy < 5%<br>Penile bulb: V29.5 Gy < 50%<br>Bowel: V18.1 Gy < 5cc, V30 Gy < 1cc
Including patients with prior RT	Ballas et al.	D30% < 18.8 Gy<br>D60% < 10 Gy	D40% < 18.1 Gy<br>D50% < 16.6 Gy	Dmax < 33.7 Gy<br>Dmean ≤ 31 Gy	Femoral heads: V14.5 Gy < 5 %,<br>Penile bulb: V29.5Gy < 50%
	Olivier et al.	V12 < 20%<br>V27 < 2cc	V12 < 15%<br>V27 < 5cc		
	Arcangeli et al.	Dmax <75% of PD		Dmax < 125% of PD	
	Scher et al.	V27 <20 cc<br>Dmax = 40.5 Gy	V19 <15cc<br>V40<5 cc		
	Jereczek-Fossa et al.	D30% < 13.5 Gy<br>D60% < 6.7 Gy	D30% < 10.6 Gy		
	Zerini et al.	mean dose to 30% of the rectal volume (DR30) < 8.4 Gy;<br>mean DR60 < 4.08 Gy	mean DB30 < 3.94Gy		
	Janoray et al.	V18.1 Gy < 50%<br>V29 Gy < 20%<br>V36 Gy < 1 cc	V18.1 Gy < 40%<br>V37 Gy < 10 cc		Testes: Blocked organ<br>Femoral heads: V14.5 Gy < 5%

PD - prescription dose. *Bladder wall – defined as outer 5 mm of bladder contour.

Table S4. Outlook current studies.

Identifier/Status	Design/Patients	Eligibility	SBRT	Primary Objectives	Secondary Objectives
NTC04915508<br>Recruiting	Prospective Phase II study<br>(102 patients, no prior RT)	Patients after RP with one of the following: Adverse pathologic fea-tures at the time of prostatectomy (positive surgical margin, pT3-4 disease, Gleason 8-10 disease, or presence of tertiary Gleason grade 5 disease), Rising PSA (at least two consecutive draws), exceeding 0.03 ng/ml, Intermediate- or high-risk Decipher genomic classifier score, >= 1 lymph node at the time of prostatectomy (pN+ disease)	SBRT every other day or on consecutive days for up to 14 days<br>Patients may receive ADT at the discretion of the treating physi-cian.	2-year GU/GI symp-toms (based on EPIC)	GU/GI symptoms (based on EPIC) at 3 months, 6 months, 1 year and 5 years<br>Acute physician scored toxicity according to CTCAE<br>5-year cumulative incidence of physician scored toxicity according to CTCAE<br>5-year PFS, bRFS, DMFS
NCT04848909<br>Not yet recruiting	Prospective Phase I study	Patients after RP: Able and willing to complete EPIC, PORPUS, and EQ-5D questionnaires,<br>A detectable PSA ≤ 2.0 ng/ml,	30 Gy in 5 fractions to the prostate bed and 25 Gy in 5 fractions to	Incidence of acute GU/GI toxicities based on CTCAE	Incidence of late GU/GI toxicities based on CTCAE

	(30 patients, no prior RT)	Two consecutive rises in PSA and final PSA > 0.1 ng/ml OR three or more consecutive rises in PSA, no N+ patients	the pelvic nodes if elective nodal irradiation is used, delivered every other business day.		Quality of Life of participants using the Expanded Prostate Cancer Index Composite questionnaire<br>Biochemical disease-free survival<br>Health Utilities using The Patient-Oriented Prostate Utility Scale<br>Health Utilities using EuroQol- 5 Dimension Questionnaire
NCT04536805<br>Recruiting	Prospective Phase I/II study<br>(44 patients, previously irradiated)	Patients after RP: Biochemical recurrence at least 2 years after EBRT and/or the end of hormone therapy, Local recurrence in irradiated areas proven by biological (PSA > 0.2 ng/ml and ascending confirmed by 2 successive assays) and radiological (lesion visible on MRI and/or Choline PET and/or PSMA PET) or histological examinations.<br>Recurrence without rectal invasion and remote recurrence from vesico-urethral anastomosis (> 10 mm) to limit urinary toxicity. Macroscopic Target Volume (GTV) ≤ 27cm3 to limit toxicity<br>Pelvic and prostate MRI evaluation + Absence of pelvic lymph node or metastatic recurrence proven by choline PET or PSMA PET scan<br>WHO performance status 0-1<br>PSA ≤ 10 ng / ml, PSA doubling time > 6 months<br>No anti-cancer treatments planned for the current relapse, including hormone therapy.<br>Age > 18 years old.<br>Life expectancy greater than or equal to 5 years	5 or 6 fractions, at a level of 5 or 6 Gy per fraction (either 5 x 6 Gy, 6 x 6 Gy, or 5 x 5 Gy), over a maximum of 12 days (from day 1 to day 10 or 12) to provide a total dose of 25 to 36 Gy<br>Oral Metformin	For phase 1: Select the recommended dose for SBRT (either 5 x 6 Gy, 6 x 6 Gy, or 5 x 5 Gy), SBRT toxicity during the 12 weeks following the initiation of SBRT<br>For phase 2: Efficacy of re-irradiation SBRT in combination with Metformin in terms of bRFS rate, PSA levels will be assessed every 3 months within 3 years after SBRT	Efficacy of re-irradiation SBRT in combination with Metformin in terms of bRFS up to 5 years<br>PFS and OS after SBRT<br>Late GU/GI toxicity after SBRT<br>Quality of life after SBRT<br>Urinary symptoms using IPSS<br>Erectile function using International Index of Erectile Function (IIEF5)
NCT04067570<br>Recruiting	Prospective single arm<br>(30 patients, no prior RT)	Patients after RP: Able and willing to complete self report questionnaires<br>Pathologic stage T3 or T4 (without any gross residual disease), NX-0, M0, and/or +ve surgical margins, and/or a rising PSA post-radical prostatectomy on at least 2 consecutive measurements	30 Gy in 5 fractions, once weekly to prostate bed, 25 Gy in 5 fractions, once weekly simultaneously to pelvic lymph nodes<br>6-24 months of androgen deprivation therapy (ADT)	Acute GU/GI toxicities based on CTCAE	Incidence of late GU and GI toxicities (≥6 months)<br>Biochemical disease-free survival (bDFS)
NCT02976402<br>Recruiting	Prospective Phase I study<br>(30 patients, no prior RT)	Patients after RP: Pathologic (p)T3 disease, positive margin(s), Gleason score 8-10, or seminal vesicle involvement<br>Undetectable post-radical prostatectomy PSA that becomes detectable and then increases on 2 subsequent measurements (PSA of > 0.1 - ≤ 2.0 ng/mL)<br>Life expectancy: 10 years<br>ECOG performance status of 0 -1<br>No distant metastases, based on the following workup within 60 days prior to registration	31 Gy in 5 sessions each of 6.2 Gy (adjuvant intent) delivered in one week,<br>32.5 Gy in 5 sessions each of 6.5 Gy (salvage intent) delivered in one week	Feasibility (ability to deliver radiation treatment as planned), Monitoring treatment related adverse events (CTCAE)	Incidence of acute and late treatment related adverse events (CTCAE)<br>Incidence of post-treatment quality of life impairment assessed through validated tools (EPIC)<br>Incidence of post-treatment abnormal laboratory values (PSA relapse)

		MRI of the pelvis PSMA/Choline PET<br>Patients can be on androgen deprivation therapy			
NCT01923506<br>Active, Not recruiting	Prospective Phase I study<br>(28 patients, no prior RT)	Patients after RP: N0 and M0 based on CT abdomen and pelvis and whole body bone scan within 120 days prior to study entry (nodes < 1.5 cm will be considered reactive and biopsy is not required; nodes ≥ 1.5 cm are required to undergo biopsy and be negative prior to study registration), bone scan findings in the absence of blastic or lytic lesion correlates on CT imaging will also be deemed non-neoplastic, ECOG 0-2<br>Prostate specific antigen (PSA) value can be undetectable up to a value of 2.0 within 30 days prior to study entry<br>PSA value that is undetectable can be enrolled if pathology from prostatectomy demonstrates one or more of the following: positive margin, extracapsular extension, or seminal vesicle invasion	Patients receive 5 fractions of SBRT over 1.5 weeks (dose escalation study)	MTD, defined as the highest dose tested in which fewer than 33% of patients experienced dose limiting toxicity (CTCAE)	Incidence of acute GU/GI toxicities based on CTCAE<br>bRFS<br>Prospective QoL data related to bowel, urinary, and sexual health
